# Supplementary material for: Sustained-input switches for transcription factors and microRNAs are central building blocks of eukaryotic gene circuits
Source: Genome Biol. 2013 Aug 23;14(8):R85. doi: 10.1186/gb-2013-14-8-r85 (PMC4054853; doi:10.1186/gb-2013-14-8-r85)
Supplement: Additional file 5 — HTML Browsable Motif Output. Zipped folder containing all WaRSwap and FANMOD motif output, viewable in a web browser. [file gb-2013-14-8-r85-S5.ZIP › HTML_browsable_motif_output/WaRSwap_all_tair9/sigs_WSWRSLDraw-2000_all.pvals.heatmaps.html/motif_id_174_010101110_tftype_all_upstream_-1000_0.html]

```
BG_MODEL = Rnew2
MOTIF_ID = 174_010101110
TF_TYPE = all
UPSTREAM = -1000_0


PVals
FN_0.2	FN_0.4	FN_0.6	FN_0.8
dg_60.genes	0.938	0.009	0.309	0.142
dg_70.genes	0.929	0.011	0.335	0.129
dg_80.genes	0.941	0.011	0.347	0.137

ZScores
FN_0.2	FN_0.4	FN_0.6	FN_0.8
dg_60.genes	-1.569	2.428	0.334	0.36
dg_70.genes	-1.508	2.381	0.241	0.445
dg_80.genes	-1.571	2.362	0.247	0.398

StDevs
FN_0.2	FN_0.4	FN_0.6	FN_0.8
dg_60.genes	1454.672	458.637	29.346	1.087
dg_70.genes	1528.602	461.664	31.18	0.979
dg_80.genes	1474.875	471.175	31.213	1.049
```
